# A Case of Charcot-Marie-Tooth Disease Causing Colitis and Electrolyte Imbalances

**DOI:** 10.7759/cureus.67918

**Published:** 2024-08-27

**Authors:** Stefany Panunzio, Lester Couch, Asm Rahman

**Affiliations:** 1 Department of Research, Alabama College of Osteopathic Medicine, Dothan, USA; 2 Department of Internal Medicine, UF Health Leesburg Hospital, Leesburg, USA

**Keywords:** diabetes mellitus, painful neuropathy, gait disturbance, peripheral motor neuropathy, functional constipation, infectious colitis, urinary tract infection, charcot-marie-tooth disease

## Abstract

Peripheral nerve injury is a result of the rare and crippling Charcot-Marie-Tooth (CMT) disease. Although it can happen at any age, progressive muscle weakening is most obvious in adolescence or the early stages of adulthood. We present a case of an 81-year-old female with recurrent urinary tract infections (UTIs), complaints of abdominal pain and constipation, as well as dysuria with abnormal electrolyte levels. This case serves as an effective symptomatic treatment plan for a patient with this rare neuromuscular disorder.

## Introduction

Charcot-Marie-Tooth (CMT) disease is a rare disorder characterized by heterogeneously transmitted peripheral neuropathy that can affect people of all ages. Patients exhibit mild to moderate sensory nerve loss in a distribution of stocking and gloving, along with distal muscle weakness and atrophy. According to recent research, CMT affects 10 to 40 people for every 100,000 people [1]. Genetic analysis has revealed that over 100 genes are present in patients with CMT, with 80-90% of these exhibiting genetic abnormalities caused by copy number variations (CNVs) in peripheral myelin protein 22 (PMP22) and mutations in the GJB1, MPZ, and MFN2 genes [2]. The quality and accuracy of numerous studies vary, which makes it challenging to determine the actual prevalence of the disease because it presents differently in various patients and has a wide range of clinical symptoms. Because Schwann cells and axons interact closely, demyelinating neuropathies in CMT frequently develop into functional axonopathies, which, in turn, cause secondary axonal degeneration [2]. Here, we report an 81-year-old female, with a known history of CMT and unable to ambulate for the past four years, who has responded well to treatment with antibiotics and electrolyte stabilization.

## Case presentation

An 81-year-old female presented to the emergency department with complaints of abdominal pain and constipation, as well as dysuria with a known history of recurrent urinary tract infections (UTIs). She was admitted to the medicine service with hypocalcemia, hyponatremia, and hypotension. Her past medical history was significant for Charcot-Marie-Tooth disease, bullous pemphigoid, type II diabetes, hypertension, hyperlipidemia, pulmonary embolism, UTIs, anxiety, paresthesia, and coronary artery disease. The patient’s medications included acetaminophen 650 mg daily, alprazolam 0.5 mg daily, aspirin 325 mg daily, atorvastatin 40 mg daily, carvedilol 6.25 mg daily, lisinopril 10 mg daily, oxycodone 15 mg daily as needed, phenazopyridine 100 mg daily, and torsemide 20 mg every other day. The patient’s family history was significant for Charcot-Marie-Tooth disease in her mother. Upon further questioning, the patient endorsed being diagnosed with CMT in her early 40s and being tested due to her significant difficulty in balance and nerve pain. She stated she initially felt mild numbness in her legs that progressed significantly and led to her being unable to ambulate for the past four years.

On physical exam, the patient appeared well but under acute distress due to abdominal pain and dysuria. On gastrointestinal exam, the abdomen was soft and non-tender, and no hepatosplenomegaly was appreciated. The skin was positive for fluid-filled vesicles in her bilateral lower extremities. On musculoskeletal exam, there was no appearance of clubbing, and upper extremity muscle mass and range of motion were within normal range. Lower extremities had loss of range of motion and the patient was not able to ambulate.

**Figure 1:**
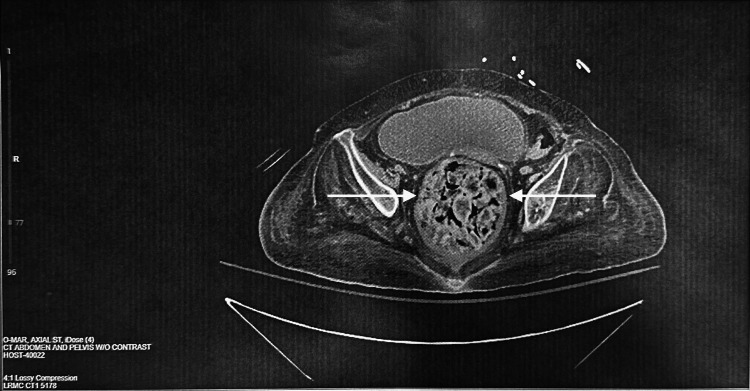
CT abdomen and pelvis w/o contrast Fecal impaction in the rectum with mild wall thickening and surrounding fat stranding, suggestive of stercoral colitis/proctitis, is seen. The moderate to large colonic stool burden is suggestive of constipation.

Gastroenterology was consulted for the patient due to abdominal pain and constipation. CT without contrast of the abdomen and pelvis was performed and revealed colitis. As shown in Figure 1, the CT scan revealed fecal impaction in the rectum with mild wall thickening and surrounding fat stranding, suggestive of stercoral colitis/proctitis. Initial laboratory work revealed a white blood cell (WBC) count of 8.0, hemoglobin (HGB) of 10.3, hematocrit (HCT) of 30.2, and Na of 132. Additionally, nephrology was consulted to evaluate electrolyte imbalances. Due to the patient’s hypotension and characteristics of sepsis, blood and urine specimens were sent for culture. In the blood specimens, no growth was found after five days. Urine cultures were negative and had no significant growth, although there was mixed flora indicating contamination.

The patient was treated with Tolvaptan tablet 15 mg for her hyponatremia and digital disimpaction was performed by the gastroenterologist to relieve constipation. The patient was placed on an IV neo-synephrine drip due to her hypotension. During her hospital course, she developed reciprocal hypertension due to withholding her blood pressure medications, and IV Nicardipine was started. She was then started on empiric IV Ciprofloxacin 400 mg/200 mL D5W for her colitis. She was also given an Aztreonam 1g injection for her UTI and sepsis. The patient’s serum sodium and calcium levels gradually improved and were then within normal limits status post tolvaptan 15 mg and calcium gluconate. Due to the bullous pemphigoid condition affecting the patients’ lower extremities, wound care was consulted. The patient was started on apixaban 2.5 mg daily due to a past medical history of pulmonary embolism.

During her hospital course, the patient was treated for colitis and her hyponatremia, hypocalcemia, and hypomagnesemia were corrected by nephrology, and the presenting symptoms improved. Due to her lack of ambulation, causing significant health problems, she was discharged home under hospice care and advised to follow up with her primary care physician in three to five days.

## Discussion

CMT is a serious disease that has a profound impact on a patient’s quality of life and affective management has limited scientific evidence. Walking can be painful and difficult for those who have Charcot-Marie-Tooth disease, especially when they're barefoot. They also have a slower-than-average gait distance and speed, and trip and fall more frequently. Clinical management geared toward improving gait is critical because these gait impairments and associated complaints are incapacitating [3]. Surgical and conservative measures are two treatment options that lack vast evidence in improving optimal patient outcomes. Demyelinating (type 1) and axonal (type 2) are the two main forms of CMT, which are primarily distinguished by their electrophysiological phenotype. Nonetheless, patients in a certain number of families exhibit traits that make it difficult to categorize them as demyelinating or axonal. As a result, these families comprise a third category of CMT patients known as "intermediate" CMT, which makes up about 10% of cases [4]. The first step to classify the neuropathy is using a nerve conduction study. This case report highlights the variable disease manifestations that CMT can provide and the complications that can present. In this case, we report an 81-year-old female with CMT who responded well to antibiotics and supportive management.

The past few years have seen a significant improvement in our understanding of the pathomechanisms of CMT as well as the yield of genetic diagnostics, owing to advancements in genetics, cellular and animal models, and genetics. However, symptomatic medication treatment, skeletal deformity surgery, and rehabilitation therapy remain the mainstays of treatment for CMT and related neuropathies while we wait for an effective therapy that can alter the natural course of the disease. Based on nerve conduction studies, CMT is divided into two categories: demyelinating and axonal varieties. Autosomal dominant inheritance demyelinating neuropathies with slowed nerve conduction velocities (NCVs) are classified as CMT1; autosomal recessive demyelinating forms are classified as CMT4; and primary axonal types with autosomal dominant or recessive transmission are classified as CMT2. There are intermediate forms of CMT with NCV values that lie between those of CMT1 and CMT2. The most notable of these is CMTX1, which is linked to GJB1 gene mutations on chromosome Xq13.1; there are also less common forms that are either autosomal dominant or recessive. The term "distant Hereditary Motor Neuropathies" (dHMNs) refers to pure motor forms of CMT that preserve sensory nerves and can be inherited autosomally, recessively, or through X-linked transmission [5].

Lower urinary tract (LUT) dysfunction has only been mentioned in a small number of reports on the documented cases of autonomic nervous system and organ dysfunction caused by CMT. These cases have never been thoroughly studied. Clinically, gastrointestinal disorders frequently accompany lower urinary tract dysfunctions and vice versa. According to animal studies, there are cross-excitatory reflexes between the colon, rectum, and bladder that are linked to several pathological conditions. It has been common to describe autonomic nervous system (ANS) dysfunction (papillary anomalies, hearing loss, dysphagia, gastrointestinal, and urinary disturbances) in individual CMT patients; however, research establishing the actual prevalence of ANS-affecting symptoms in these patients is lacking [6]. There have also been reports of dysautonomia features in CMT, such as orthostatic hypotension, hyperhidrosis, and urgency and incontinence of urine. Individuals who have INF2 mutations may experience focal segmental glomerulosclerosis, a condition that can quickly lead to end-stage renal disease [2]. In this case, the patient's initial blood urea nitrogen (BUN) of 42 mg/dL was increased to 50 mg/dL during her hospital stay, indicating mild kidney dysfunction.

Diabetes mellitus (DM) is a prevalent metabolic disease that is exemplified by persistently elevated blood sugar levels, which may be linked to microvascular complications. Although coexisting CMT and DM are both fairly common, there have rarely been reports of these two conditions happening at the same time. According to reports, CMT1A coexisting with DM has the potential to exacerbate the neurophysiological and clinical manifestations of the CMT1A phenotype [7]. The patient's history of type II diabetes concurrently with CMT exemplifies the clinical importance of treatment management of patients with these concurrent diseases. With the patient’s family history of CMT in her mother, concurrently with developing type II diabetes, her clinical presentations and hospitalizations were further complicated by the exacerbations of her disease pathophysiology.

Finding therapeutic targets that may be used widely is of great interest because various forms of CMT share similar pathological pathways. The most promising finding to date to come out of these efforts is the landmark identification of Sarm1 as a critical gene in axonal degeneration. Sarm1 is a NADase that causes NAD+ to be depleted, which initiates metabolic signaling processes that lead to axonal degeneration. While it is not currently possible to remove the disease-causing allele selectively while leaving the wild-type allele intact, some groundbreaking research highlights the potential of mutant allele-specific genetic silencing. In mouse models, allele-specific siRNA has been successful in targeting two different GARS mutations that cause CMT2D [8]. However, new insights into allele-specific curative therapies are provided by developments in gene replacement therapy. But, to identify any potential benefit from these treatments, it is crucial to properly plan clinical trials and create relevant biomarkers, especially when the disease progresses slowly, as in the case of CMT1A patients [9].

A distinct therapeutic challenge arises for individuals with CMT. The cornerstone of CMT2A treatment up to this point has been supportive care, the most significant component of which is physical activity. Hydrotherapy, energy-saving methods, fall risk prevention tactics, mobility training, physical rehabilitation, stretching, posture stability, patient education, training on proper assistive devices, and prevention of secondary impairments are important aspects of physical therapy and rehabilitation for patients with CMT. In the treatment of CMT, protecting the joint range of motion (ROM) to minimize functional use of all extremities and prevent the risk of contractures should be emphasized [10]. By the time the patient presented to the emergency department, she had been managed conservatively by her primary care physician to treat her symptoms of pain and muscle weakness. When the patient presented with secondary manifestations of the disease such as constipation, UTI, and electrolyte imbalances, due to her nervous system disorder, appropriate care was given to stabilize the patient and return her to her baseline functions. A personalized medicine approach with disease-specific therapies is necessary to address the various mechanisms of CMT neuropathies. Additionally, axonal degeneration's final common pathways represent a major therapeutic target. This is a very active field with several CMT forms currently undergoing preclinical and clinical trial stages of novel treatment approaches. The development of more thorough clinical evaluation tools, the identification of disease biomarkers that may respond to treatment, and the improvement of clinical trial design have all been prompted by the lessons learned from early CMT1A clinical trials [11].

## Conclusions

CMT presents various obstacles in treatment. While there are ongoing treatments available, it still stands to be a disease with symptomatic treatment that requires attention to various organ systems. Due to each patient presenting with various symptomatic pathophysiology, this disease has a long line of therapies to be further discovered. Here, we report a case of aggressive administration of IV ciprofloxacin, aztreonam, and IV Samsca in an 81-year-old female, leading to the resolution of her presenting symptoms.
